# Structure-Based Discovery of Dual-Target Hits for Acetylcholinesterase and the α7 Nicotinic Acetylcholine Receptors: In Silico Studies and In Vitro Confirmation

**DOI:** 10.3390/molecules25122872

**Published:** 2020-06-22

**Authors:** Sebastian Oddsson, Natalia M. Kowal, Philip K. Ahring, Elin S. Olafsdottir, Thomas Balle

**Affiliations:** 1 Faculty of Pharmaceutical Sciences, School of Health Sciences, University of Iceland, 107 Reykjavík, Iceland; seb20@hi.is (S.O.); nmp@hi.is (N.M.K.); elinsol@hi.is (E.S.O.); 2 Sydney School of Pharmacy, Faculty of Medicine and Health, The University of Sydney, Sydney, NSW 2006, Australia; philip.ahring@sydney.edu.au; 3 Brain and Mind Centre, The University of Sydney, Camperdown, NSW 2050, Australia

**Keywords:** high-throughput virtual screening, dual-target lead discovery, neurodegenerative disorders, Alzheimer’s disease, dual mode of action, multi-modal, nicotinic acetylcholine receptor, acetylcholinesterase, molecular docking

## Abstract

Despite extensive efforts in the development of drugs for complex neurodegenerative diseases, treatment often remains challenging or ineffective, and hence new treatment strategies are necessary. One approach is the design of multi-target drugs, which can potentially address the complex nature of disorders such as Alzheimer’s disease. We report a method for high throughput virtual screening aimed at identifying new dual target hit molecules. One of the identified hits, *N*,*N*-dimethyl-1-(4-(3-methyl-[1,2,4]triazolo[4,3-a]pyrimidin-6-yl)phenyl)ethan-1-amine (Ýmir-2), has dual-activity as an acetylcholinesterase (AChE) inhibitor and as an α7 nicotinic acetylcholine receptor (α7 nAChR) agonist. Using computational chemistry methods, parallel and independent screening of a virtual compound library consisting of 3,848,234 drug-like and commercially available molecules from the ZINC15 database, resulted in an intersecting set of 57 compounds, that potentially possess activity at both of the two protein targets. Based on ligand efficiency as well as scaffold and molecular diversity, 16 of these compounds were purchased for in vitro validation by Ellman’s method and two-electrode voltage-clamp electrophysiology. Ýmir-2 was shown to exhibit the desired activity profile (AChE IC_50_ = 2.58 ± 0.96 µM; α7 nAChR activation = 7.0 ± 0.9% at 200 µM) making it the first reported compound with this particular profile and providing further evidence of the feasibility of in silico methods for the identification of novel multi-target hit molecules.

## 1. Introduction

The development of treatments for neurodegenerative disorders such as Alzheimer’s disease (AD), the most prevalent type of dementia, is a pressing matter due to the incurable, progressive, and debilitating nature of the disease [1]. The situation is further aggravated by the ageing of populations worldwide and reflected by the estimated triplication of the AD-affected from 50 million in 2018 to 152 million in 2050 [2]. Neuropathologically, AD is characterized by its accompanying lesions, most notably but not exclusively by amyloid plaques, neurofibrillary tangles, and neuronal and synaptic loss [3,4], that lead to a variety of neurochemical changes, prominently in the cholinergic system [5]. Taken together, these alterations are the cause of cognitive symptoms such as memory loss, language impairment, visuospatial dysfunction, and executive functioning issues [6] which are often accompanied by other behavioral and psychological symptoms (BPSD) [7,8].

**Figure 1:**
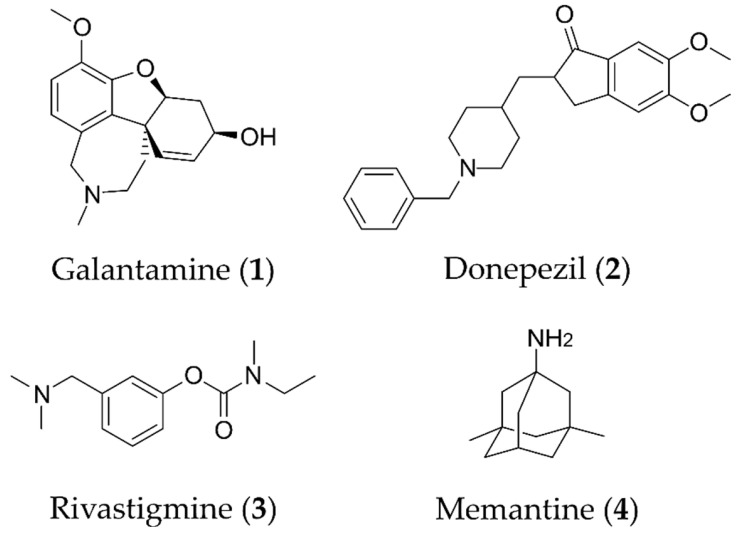
Structures of currently FDA-approved drugs for the treatment of AD, galantamine (**1**), donepezil (**2**), rivastigmine (**3**) and memantine (**4**). Donepezil and memantine are also approved as a combination therapy.

Despite extensive research efforts to understand AD, its causes and the underlying disease mechanisms remain poorly understood. This renders the search for effective drugs difficult. AD treatments have yet to succeed in slowing down disease progression and AD medications currently approved in the US are palliative in nature but nevertheless continue to provide the biggest benefits for patients [9]. The drugs fall into two categories, acetylcholinesterase (AChE) inhibitors (galantamine (**1**), donepezil (**2**), rivastigmine (**3**); Figure 1) that raise synaptic acetylcholine (ACh) levels and memantine (**4**), an NMDA receptor antagonist that regulates glutamatergic activity [10,11].

Investigations into the effects of administering memantine in conjunction with AChE inhibitors started about two decades ago [12,13] and led to the approval of Namzaric, which is a combination therapy relying on fixed doses of donepezil and memantine [14]. Other combination therapies are also of interest, e.g., drugs with pro-cognitive effects combined with anti-inflammatory drugs [15]. In fact, treatments that target more than one disease mechanism have become an emergent therapeutic strategy, especially for the treatment of complex, multifactorial diseases [16]. Besides addressing their intricate nature, the combination of two molecules with different activities in one drug formulation can result in drug synergism, as well as prevent unwanted compensatory mechanisms and drug tolerance, for instance [17].

An alternative to combination therapies is multi-modal compounds [18,19]. These compounds have essentially the same advantages as drug combinations but have, analogously to single molecule therapies, an easier therapeutic regimen, fewer drug-drug interactions and no differences in pharmacological properties to be considered [17,19]. However, the search for multi-target ligands also poses new challenges [20]. This conundrum is commonly tackled by the synthesis of bivalent hybrid ligands [21], but computational approaches can also be used to screen for novel ligands, i.e., by exploiting pharmacophore models underlying a set of known reference ligands or models of the corresponding binding sites [22]. Virtual screening (VS) methods allow for the screening of large compound databases against these models within a reasonable timeframe at comparably low cost. A prerequisite is the availability of enough data of adequate quality in order to construct predictive models. With the number of published X-ray structures, ligand datasets, and the ever-increasing size of screening compound databases, these strategies are becoming increasingly more feasible [23]. Nevertheless, VS for multi-target-directed ligands is not as commonly used as might be expected due to the complexity of the task and a lack of established protocols [23].

In previous work, we embarked on the search for AChE inhibitors that concurrently increase receptor activation at pro-cognitive nicotinic acetylcholine receptors (nAChRs) in a subtype-specific manner [24]. In addition to the already established benefits of increasing synaptic ACh levels by AChE inhibitors, the activity profile would thus extend to direct modulation of the cholinergic system. Since the dosage of AChE inhibitors is often limited due to adverse effects, boosting the activity of a pro-cognitive receptor such as the α7 nAChR [25] could increase the overall treatment efficiency. Importantly, we established the feasibility of VS for the identification of compounds targeting AChE as well as the α7 nAChR [24]. Top-scoring VS hits obtained from one target were subsequently screened against the second target, which allowed for the identification of dual-activity compounds, however, the disadvantage of this approach was introduction of bias towards the first target screened and that none of these screening hits showed agonistic activity at the α7. Therefore, in the present study, the size of the screening database was increased ~45 fold by shifting the focus from natural products and their derivatives to drug-like molecules. Furthermore, the entirety of the screening database was screened against both targets individually. Donepezil and a recently published α7 nAChR agonist were selected as reference compounds and structure-based, parallel and independent VS was performed. Here, we present the new protocol along with in silico and in vitro results, which show that it is feasible to identify multi-target compounds with the desired activity profile.

## 2. Results

**Figure 2:**
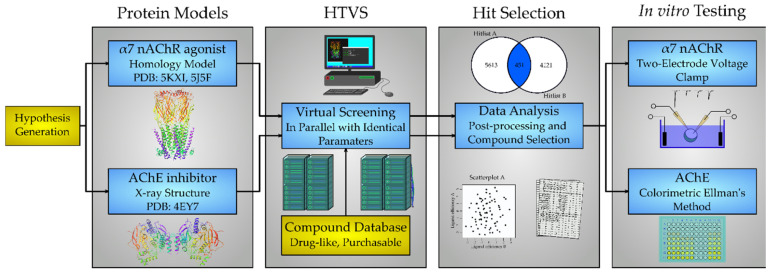
Workflow of the parallel HTVS, hit selection and in vitro evaluation. Two protein targets were selected for in silico studies and protein models suitable for docking prepared. The same database of compounds was then individually screened against each model using identical parameters. After post-processing, common compounds from the two independent screening hit lists were used to identify compounds destined for in vitro testing.

Our goal was to find new compounds with dual activity: (i) inhibitors at the AChE and (ii) agonists of the α7 nAChR as illustrated in Figure 2. The approach taken was to screen a pre-filtered compound database against an AChE X-ray structure and an α7 nAChR homology model in parallel using identical parameters. The intersecting set between the two independently obtained sets of VS hits was considered to contain potential bimodal compounds as illustrated by the Venn diagram in Figure 2. A subset of these compounds were selected for in vitro testing at human α7 nAChR expressed in *Xenopus laevis* oocytes by two-electrode voltage-clamp electrophysiology and at the human recombinant AChE using Ellman’s colorimetric method [26] to validate the in silico predictions.

### 2.1. Protein Structures and Homology Modeling

**Figure 3:**
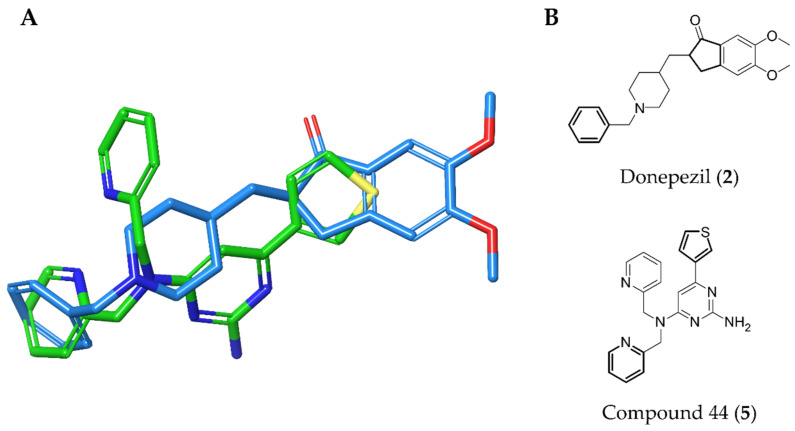
(**A**) Superposition of reference ligands in their bioactive, co-crystallized conformations. AChE inhibitor Donepezil from PDB 4EY7 is shown in blue and α7 nAChR agonist Compound 44 from PDB 5J5F in green. Atoms of the rings shown in bold in Panel B were used for superimposition of the ligands. (**B**) Chemical structures of Donepezil and Compound 44.

Based on structure-activity considerations for AChE inhibitors and α7 nAChR agonists, an X-ray structure of AChE co-crystallized with donepezil (**2**) determined to a resolution of 2.35 Å (PDB 4EY7) was selected for the high-throughput virtual screening (HTVS) [27]. Since the structure of α7 nAChR has not been determined to date, a homology model was constructed using an (α4)_2_(β2)_3_ nAChR structure with a resolution of 3.94 Å (PDB 5KXI) [28] as primary template augmented with an additional α4 subunit to facilitate modelling of an α7 homopentamer. In addition, an acetylcholine binding protein (AChBP) from *Lymnaea stagnalis* co-crystallized with *N*4,*N*4-bis[(pyridin-2-yl)methyl]-6-(thiophen-3-yl)pyrimidine-2,4-diamine (Compound 44; α7-pEC_50_ = 6.3) from a recently published ligand series (PDB 5J5F) [29] served to define and bias the binding site. This structure was chosen because donepezil is an extended, linear structure like Compound 44 (**5**), as illustrated in Figure 3. We hypothesized that including the AChBP structure with its co-crystallized ligand as an additional template in the model building process would result in an α7 nAChR model, in which the binding pocket would be opened up in a way that would facilitate the docking of extended molecules resembling donepezil in size and shape, thus enhancing the probability of identifying dual active molecules.

**Figure 4:**
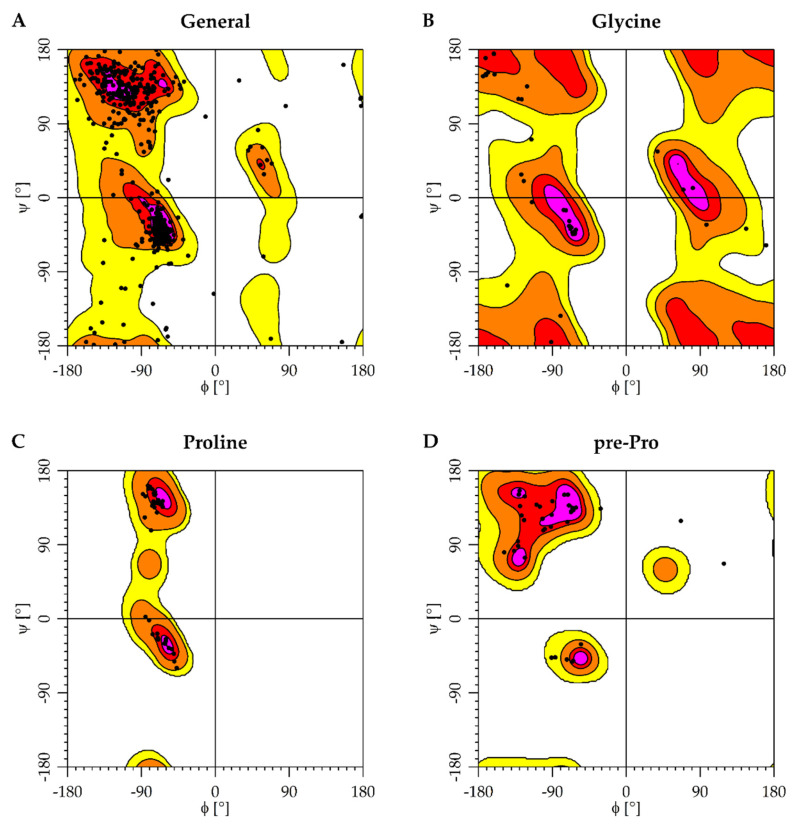
Detailed Ramachandran diagram of chains A and B of the α7 nAChR homology model by residue type. (**A**) The general case of non-glycine, non-proline and non-pre-proline residues is depicted. The special cases having either significantly less (**B**; Glycine) or more conformational restraints (**C**,**D**; Proline and preproline). The contours of Glycine are twofold symmetrized based on the dataset from Lovell et al. [31].

We selected the homology model with the lowest Discrete Optimized Protein Energy (DOPE) score [30] and inspected its Φ, Ψ angle distributions (Figure 4). A small fraction of the modelled amino acid residues occupies energetically unfavorable regions of the plot. Since all these residues are in non-conserved loop regions of the protein distant from the binding site, the model was deemed suitable for docking. Both the α7 nAChR model and AChE structure were validated by docking the respective reference ligands (RMSD Compound 44 = 0.87 Å, Donepezil = 0.62 Å).

### 2.2. Screening Database

**Figure 5:**
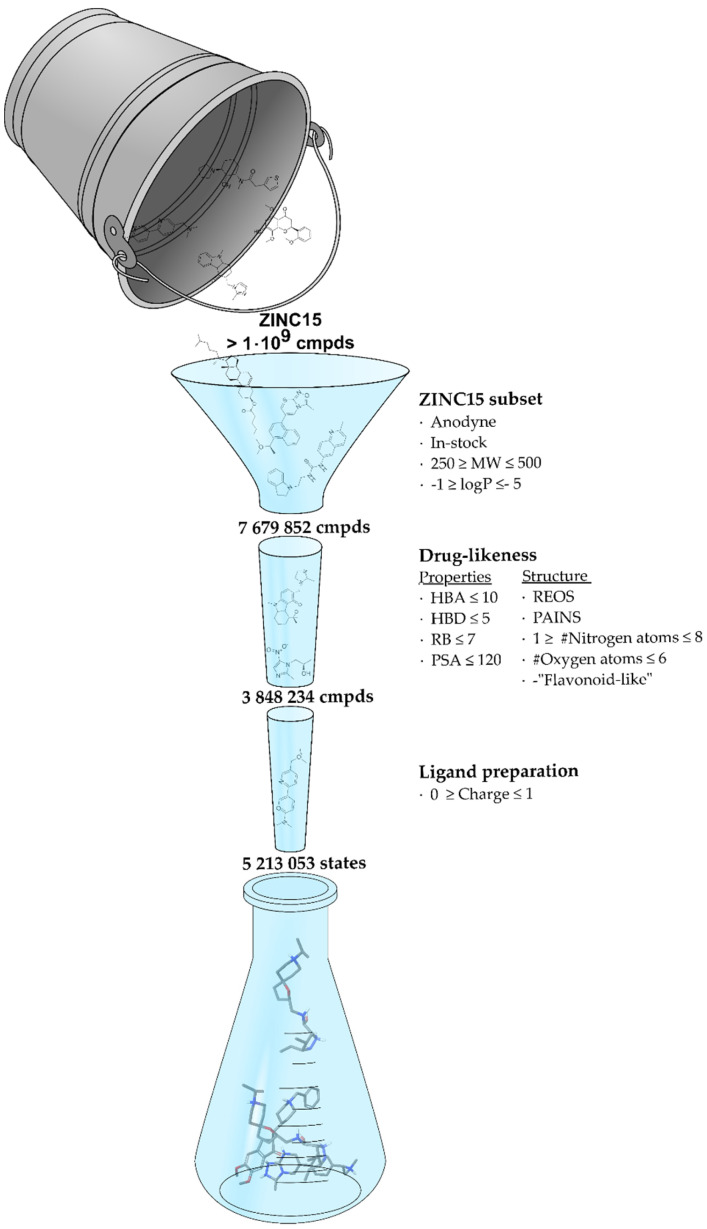
Schematic overview depicting the number of molecules during database preparation, starting from the compound database ZINC15 (bucket) through various filtering stages (funnel) to the final database used for VS (flask). States refers to the number of different entities for which 3D coordinates were generated and includes protonation states and tautomers.

To optimize the chances of success for the VS, we considered the importance of various factors that concern compounds making up the screening database. The ZINC15 database [32], which is freely available and suited for VS, provides compound annotations, such as different degrees of commercial availability and the ability to download specific subsets of the database based thereon, and was chosen as starting point. In a first step, a subset of 7,679,852 compounds was downloaded from ZINC15 (Figure 5) that corresponds to compounds which are “anodyne” (lowest reactivity), “in-stock” (highest commercial availability), have molecular weights (MWs) between 250 and 500 Da and a logP within the range of −1 to 5. This puts an emphasis on drug-likeliness and on the ability to verify the results in the laboratory. In addition to the property filters built into ZINC15, another set of property and structure filters was applied. The property filters were based on Lipinski’s rule of five [33] with the intention of enhancing the drug-likeliness of the compounds while the structural filters were more focused on reducing the probability of identifying reactive, assay-interfering or otherwise problematic ligands as exemplified by REOS (rapid elimination of swill) [34] or PAINS (pan-assay interference compounds) [35] filters. Roughly half of the initial set was discarded during this extensive filtering process. To finalize the screening database, the remaining 3,848,234 compounds were prepared for docking resulting in 5,213,053 entities. At this stage, the total charge per molecule was limited to 0 and 1 predicated on the knowledge that nicotinic receptor ligands in most instances contain a basic nitrogen atom crucial for binding.

### 2.3. Virtual Screening

**Figure 6:**
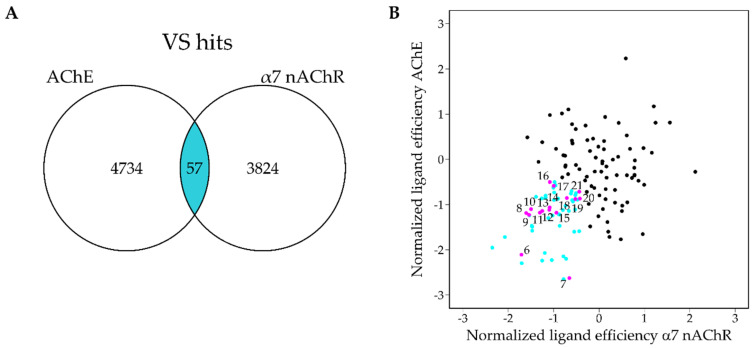
Venn diagram of AChE and α7 nAChR screening hits in the 0.5 quantile of normalized ligand efficiencies. (**B**) Scatterplot of normalized, strain-corrected ligand efficiencies. Compounds which are in the 0.5 quantile are colored blue and tested compounds are labeled as indicated Table 1 and shown in magenta.

The database was docked against both protein targets in parallel using the glide-based Schrodinger virtual screening workflow and identical parameters. Following three rounds of docking exercises, the number of compounds was narrowed down to approximately 15,000 for each target. After post-processing, the compounds below the 0.5 quantile of the normalized ligand efficiencies were considered reasonable VS hits for each target and the intersect between the two VS screening results was then investigated in more detail (Figure 6). The median set of the putative ligands consisted of 4734 and 3824 compounds for AChE and α7 nAChR, respectively, with 57 compounds shared between the two sets. The docking poses and the structural diversity of these compounds were then analyzed in order to select 15 compounds for in vitro testing. Notably, all compounds contained a basic nitrogen and at least one aromatic ring. Several hits were analogous structures, hence compounds were prioritized for in vitro testing based on the ligand efficiency values from docking and the observed molecular interactions in the docked ligand poses such as hydrogen bonding to TRP-149 of the α7 nAChR, while also ensuring that no close derivatives were present in the final set.

### 2.4. AChE and nAChR α7 Activity Testing

The selected compounds (**6**–**21**, Table 1) were screened for AChE inhibitory activity at 200 µM by employing Ellman’s colorimetric analysis [26]. For five poorly soluble compounds, the methanol supernatants were tested. Ellman’s colorimetric analysis is based on the breakdown of acetylthiocholine (ATCI) by AChE to thiocholine and acetic acid. Ellman’s reagent in turn reacts with the thiol group of thiocholine resulting in a yellow color indicating the extent of enzymatic activity. Of the eleven compounds soluble in MeOH, ten were shown to inhibit AChE significantly at 200 µM varying from 66.6–100% while one compound (**17**), showed low inhibition (22.9 ± 1.1%, Table 1), which is in agreement with it having the lowest docking score for AChE of all the compounds tested. The five poorly soluble compounds also exhibited significant inhibitory activities between 45.9 and 100%. Overall, fourteen compounds inhibited AChE enzymatic activity more than 60%, eleven more than 80% and eight more than 90%. Compounds that inhibited AChE more than 80% in the initial screening at 200 µM had IC_50_ values in the low micromolar range between 1.10 and 33.47 µM, which is two to three orders of magnitude lower than donepezil, the reference ligand, which showed an IC_50_ value of 0.06. µM. The IC_50_ of compounds **6** and **15**, the least potent AChE inhibitors, were determined to be 58.47 and 114.70 µM.

**Figure 7:**
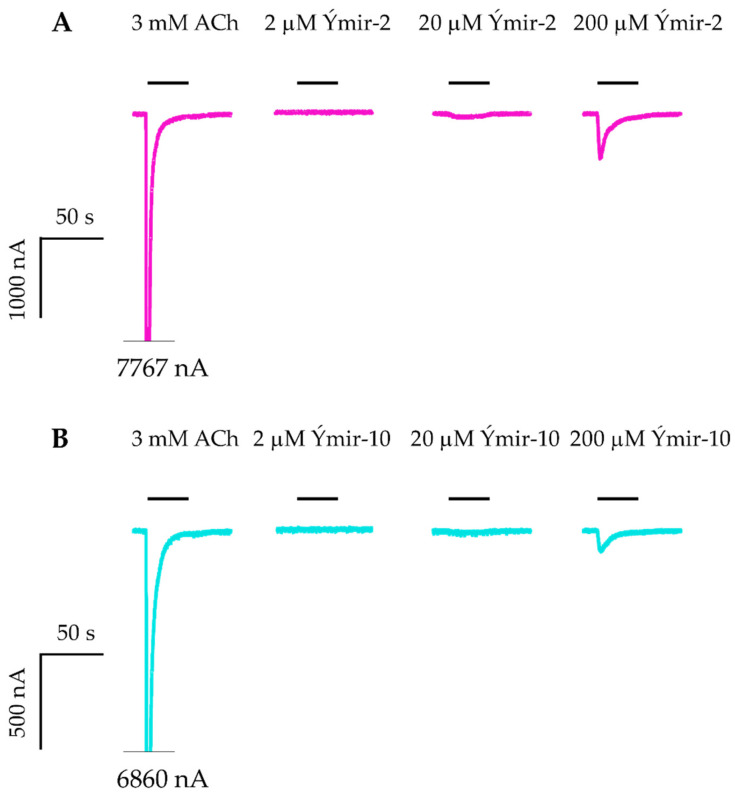
Evaluation of compounds as agonists. Representative current traces for ACh, Ýmir-2 (**7**) (**A**) and Ýmir-10 (**15**) (**B**) at α7 nAChRs expressed in *Xenopus* oocytes. Cells were subjected to two-electrode voltage-clamp electrophysiology experiments where the oocyte membrane potential was clamped at −60 mV. The representative traces were baseline subtracted. Bars above the traces represent the application periods and the respective test solution concentrations are indicated above them. Note that the majority of the washing periods (3 min) between each trace are omitted in the figure.

**Figure 8:**
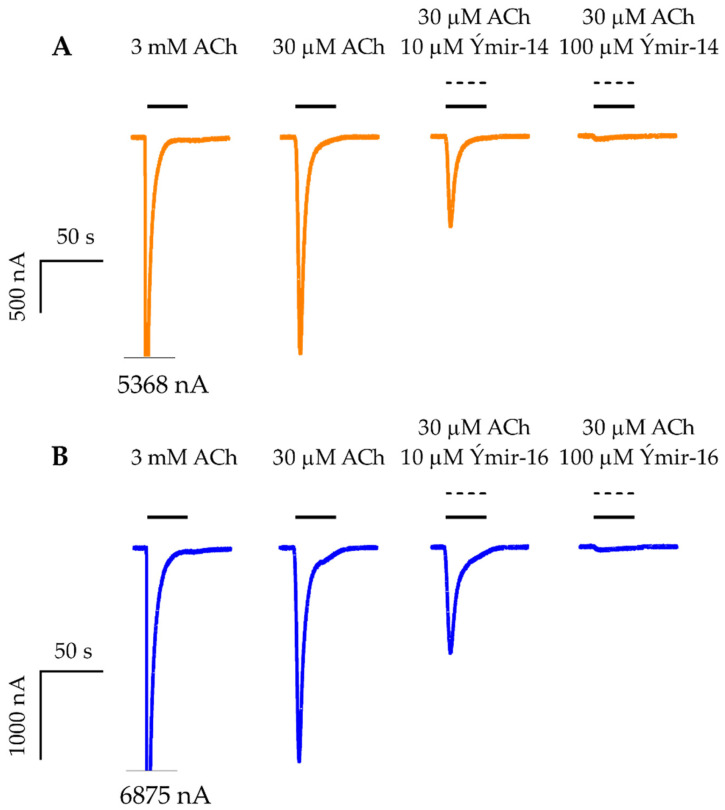
Evaluation of compounds as antagonists. Representative current traces for ACh and 10 and 100 µM Ýmir-14 (**19**) (**A**) and Ýmir-16 (**21**) (**B**) co-applied with 30 µM ACh at α7 nAChRs expressed in *Xenopus* oocytes. Cells were subjected to two-electrode voltage-clamp electrophysiology experiments where the oocyte membrane potential was clamped at −60 mV. The representative traces were baseline subtracted. Bars above the traces represent the application periods and the respective test solution concentrations are indicated above them. Note that the majority of the washing periods (3 min) between each trace is omitted in the figure.

Subsequently, all compounds except compound **17** were assessed at the α7 nAChR expressed in *Xenopus* oocytes using two-electrode voltage-clamp electrophysiology. Compounds were tested for direct activation of the α7 nAChR in a 0.2–200 µM concentration range. Compounds displaying less than 1% direct activation were further evaluated at 100 µM for their ability to alter currents evoked by 30 µM ACh. Compound **7** (Ýmir-2) and **15** (Ýmir-10) exhibited activation of α7 nAChR with 7.0 ± 0.9% and 2.3 ± 0.4% at 200 µM, respectively (Figure 7). Attempts to establish their potency were unsuccessful due to limited solubility. However, application of 2, 20, and 200 µM, as evident from Figure 7, established a concentration dependent effect. The remaining thirteen compounds exhibited less than 1% agonism indicating that they were either inefficient at mediating receptor activation or inactive at the tested concentrations. When tested as antagonists for their ability to inhibit ACh-evoked currents, all compounds showed inhibition in a range of 47.2–97.3% at 100 µM, with compounds **19** (Ýmir-14) and **21** (Ýmir-16) displaying almost full inhibition of 96% and 97%, respectively, at 100 µM (Figure 8).

## 3. Discussion

We embarked on the search for bimodal compounds with the help of computational methods. In accordance with the hypothesis from our previous study [24], we searched for hit molecules that target α7 nAChR as agonists and AChE as inhibitors. A drug based on this new activity profile could provide a new strategy for treating AD by the dual modulation of cholinergic signaling.

Despite the requirements of VS for high quality models of binding sites and screening databases, it has proven useful for the identification of new ligands for single targets and many methodological improvements have been made over the past decades [36,37]. Adding a second biological target adds another significant constraint to the problem, which is often addressed by pre-filtering or pre-screening the compound database based on one target before testing the second target [23].

In the current study, we conducted a VS without pre-screening our ligand database and docked the entire dataset to both targets. AChE and α7 nAChR are structurally and functionally distinct proteins but both evolved to accommodate ACh in their respective binding pockets. Sharing the same endogenous ligand and hence pharmacophoric elements should increase the probability of finding a molecule that fits both pockets. In addition, we constrained the search to ligands that are extended and linear based on two reference ligands.

We successfully employed this HTVS approach and identified two compounds (Ýmir-2, Ýmir-10) that showed AChE inhibition and activation of the α7 nAChR, confirming the feasibility of VS for the search of bimodal compounds at these targets as reported previously [24]. We observed a remarkably high hit rate for AChE inhibitors, where all but one (**17**) of the tested compounds showed activity at 200 µM. Moreover, 10 out of the 11 compounds soluble in MeOH showed significant inhibition of the enzymatic activity of AChE, as did all the compounds where only the supernatants were tested due to low solubility, indicating that these compounds also interact with the active site of AChE. However, further analysis to determine IC_50_ values could not be performed for these compounds.

The hit rate at the α7 nAChR was likewise high, with 2/15 compounds displaying direct agonism. Ýmir-2 (**7**) and Ýmir-10 (**15**) showed partial activation of 7.0% and 2.3%, respectively, at 200 µM. Due to solubility issues, the maximal receptor activation could not be determined. The remaining compounds exhibited α7 nAChR antagonism, in a range between 47.2% and 97.3% at 100 µM when co-applied with 30 µM ACh. Two compounds, Ýmir-14 (**19**) and Ýmir-16 (**21**) inhibited the response of ACh almost fully and in a concentration dependent manner (Figure 8). This, and the fact that the agonist-based VS project yielded compounds that are structurally similar to each other as well as to known α7 nAChR agonists, suggests that the investigated compounds interact with the binding pocket where they likely act as antagonists although weak partial agonism cannot be ruled out. Hence, as all compounds but one displayed some activity at the nAChR, and as the difference between an antagonist and agonist is often subtle, the VS in terms of binding to the α7 nAChR orthosteric binding site could be considered as high as 14/15. However, it cannot be excluded that receptor inhibition for some compounds is mediated by a different mechanism such as direct blockage of the ion channel pore. Further experiments would be required to elucidate the exact mechanism of inhibitory interaction.

**Figure 9:**
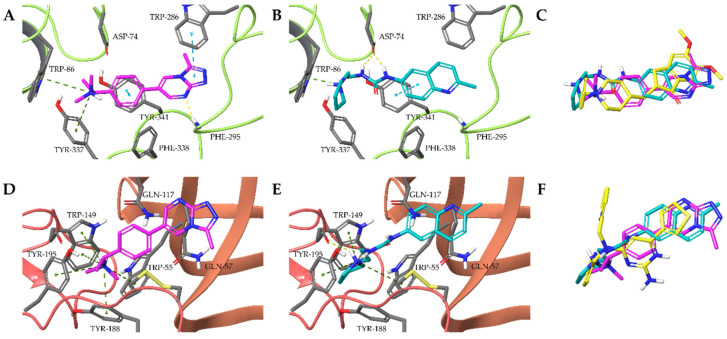
Interactions of Ýmir-2 (**7**) (magenta) and Ýmir-10 (**15**) (blue) in AChE (**A**,**B**) and α7 nAChR (**D**,**E**) and superposition of docking poses to reference ligands donepezil (**C**) and Compound 44 (**5**) (**F**). Hydrogen-bonding and is indicated by yellow, dotted lines, cation-π interactions by green, dotted lines and π–π interactions by blue dotted lines.

All 57 compounds identified in both screenings were extended, linear molecules. Other common properties such as the presence of a basic nitrogen at one end of the molecule and an aromatic moiety at the other were also observed. As the set included derivatives of the same compound but also compounds that differed only in their basic amine (e.g., piperidine and pyrrolidine) we identified these two regions as the main source of variability in the set of putative bimodal compounds. The low diversity of the intersecting set is likely a consequence of the protocol and the constraints applied during docking, but the structural patterns are also in accordance with known nicotinic ligands indicating that this is a general feature of nAChR ligands. The docked poses of Ýmir-2 (**7**) and Ýmir-10 (**15**) display characteristic interactions of AChE inhibitors and nicotinic receptor ligands around the basic amine, i.e., the positively charged amines are coordinated in the aromatic cage of the α7 nAChR and the anionic site of AChE (Figure 9). The lack of hydrogen bonds and strong hydrophobic interactions distal to the basic amines in the α7 nAChR suggests that the activity of these ligands could be further improved in this region. It is also noteworthy that the basic amine of Ýmir-2 (**7**) does not display the hydrogen bond to the backbone carbonyl of TRP-149 which is often considered crucial [38] and observed in multiple co-crystallized complexes between ligands and nicotinic receptors as well as acetylcholine binding proteins.

## 4. Materials and Methods

### 4.1. Materials

Plant origin galantamine hydrobromide analytical standard was purchased from PhytoLab GmbH & Co. KG (Vestenbergsgreuth, Germany). Screened compounds (**6**–**16**, **18**, **20** and **21**) were purchased from Molport (Riga, Latvia), compound **17** from Asinex (Winston Salem, NC, USA) and **19** from AsisChem (Waltham, MA, USA). Restriction enzymes were from New England Bio Labs Inc. (Ipswich, MA, USA) and DNA and RNA purification kits were from QIAGEN N.V. (Venlo, The Netherlands). mMessage mMachine T7 transcription kit was from ThermoFisher Scientific (Waltham, MA, USA). Human recombinant acetylcholinesterase (C1682), acetylcholine chloride, acetylthiocholine iodide, 5,5-dithiobis-(2-nitro-benzoic acid), bovine serum albumin, kanamycin, theophylline, tricaine, collagenase, HEPES, Trizma, salts and other chemicals not mentioned specifically were purchased from Sigma-Aldrich Co. LLC (St. Louis, MO, USA) and were of analytical grade.

### 4.2. Protein Models

#### 4.2.1. nAChR α7 Homology Model

**Figure 10:**
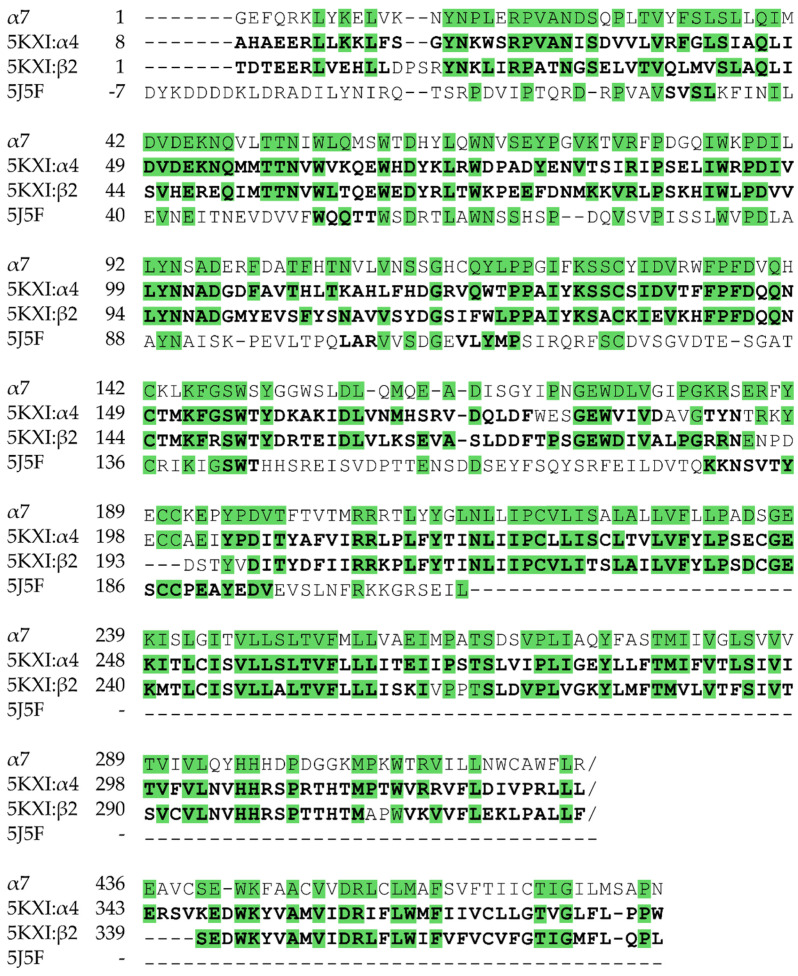
Sequence alignment of the templates used in the homology model of α7 nAChR. The sequence of one α- and one β-subunit of the (α4)_2_(β2)_3_ receptor co-crystallized with nicotine (PDB 5KXI) and of an AChBP co-crystallized with Compound 44 (**5**) were aligned using T-Coffee and modified for homology model building. Residues used as templates in homology modelling are shown in bold and conserved amino acids highlighted green. The overall sequence identity between the α7 nAChR and the (α4)_2_(β2)_3_ nAChR subunits used as primary templates are 45% (62% sequence similarity) and 37% (57% sequence similarity), respectively. The sequence identity between α7 nAChR and AChBP is 28% (46% sequence similarity) overall and 39% (64% sequence similarity) for the part that was used as template.

The amino acid sequence of the human α7 nAChR was obtained from the UniProt protein knowledgebase (entry P36544 [39]) and aligned to the sequences of the α4- and β2-subunits of PDB 5KXI [28] (α- and β-subunits or chains A and B respectively) and 5J5F [29] (chain A) with the T-Coffee Expresso structural alignment tool [40]. Residues used to build the homology model were then manually selected ensuring that the binding site was mainly built on the 5J5F template, an AChBP structure in complex with Compound 44 (**5**) (*N*4,*N*4-bis[(pyridin-2-yl)methyl]-6-(thiophen-3-yl)pyrimidine-2,4-diamine), whereas the tertiary and quaternary structure was largely modelled after 5KXI (Figure 10). Since the α7 receptor consists of α-subunits only, a single α-subunit from the (α4)_2_(β2)_3_ template was also provided separately as template where there is a β-subunits in the 5KXI complex. The α7 nAChR homology models were built with Modeller (Version 9.16, Automodel class, Salilab, UCSF, San Francisco, CA, USA) [41]. Protein secondary structures as well as the pentameral symmetry were supplied as constraints to Modeller. Out of 100 generated models, the model with the highest scoring DOPE potential [30] was selected for further studies.

The resulting receptor model was then prepared with the Schrodinger Protein preparation wizard [42] (hydrogens added and H-bonds optimized, protonation according to pH 7.4, restrained minimization (RMSD 0.3 Å)). The quality of the selected model was assessed based on a Ramachandran plot drawn in R [43] using the data published by Lovell et al. [31]. Subsequently, the protein-ligand complex was refined with Schrodinger Prime [44] for the ligand its surrounding protein residues within 5 Å using Monte Carlo sampling [45], VSGB solvation model [46], OPLS3 force field [47], and otherwise default parameters.

#### 4.2.2. AChE Model

The protein structure of AChE (PDB 4EY7 [27]) was downloaded from the Protein Data Bank [48] and all waters, ions, and small molecules except for donepezil removed. It was then prepared with the Schrodinger Protein preparation wizard [42] as described above for the α7 nAChR model.

### 4.3. Screening Library

The ZINC15 database [32] was downloaded in smiles format after application of the following filters: MW 250–500; logP −1–5; in-stock; anodyne. The smiles strings were then converted to canonical smiles and duplicates removed using OpenBabel 2.4.0 [49]. REOS [34] and PAINS [35] structure filters were applied using Schrodinger utilities. A set of additional filters were defined and applied to remove “flavonoid-like” structures, restricting the amount and position of halogen atoms and limiting the number of oxygen and nitrogen atoms to 6 and 1–8, respectively. Finally, physicochemical properties were calculated using Schrodinger software (Release 2018-1, Schrödinger, LLC, New York, NY, USA) and filtered using a custom perl script (HBD 5; HBA 10; PSA ≤ 120, RB ≤ 7).

### 4.4. Virtual Screening

With respect to VS, 14 Å^3^ Glide Receptor Grids [50,51] were centered around donepezil and Compound 44 in the AChE and α7 nAChR protein models respectively and generated with the receptor scaling factor set to 0.9, the partial charge cut off to 0.3 and three positional restraints along the reference ligands in each of the binding sites. For the α7 nAChR, the areas concerned were in the hydrophobic pocket as well as the area corresponding to the position of the thiophen moiety of the *N*4,*N*4-bis[(pyridin-2-yl)methyl]-6-(thiophen-3-yl)pyrimidine-2,4-diamine ligand in the 5J5F structure and for AChE they were set at the anionic site as well as the hydrophobic gorge occupied by the indanone moiety of donepezil. These positional restraints were applied after each round of docking, thereby ensuring that only ligand poses occupying crucial areas in the binding site were considered without artificially enriching them. To perform the Ligand Docking, the Virtual Screening Workflow available in the Schrodinger suite based on Glide docking [50,51] was used. Ligands were prepared with OPLS_2005 forcefield [52] at pH 7.4 and a maximum of 8 different stereoisomers per ligand generated. Only neutral and mono charged states were kept (See Section 4.3 also). For docking, the ligand scaling factor set to 0.85 and the partial charge cut-off to 0.2. Moreover, 300,000 compounds were kept after the initial HTVS, 30,000 after the SP (Standard Precision), and none were removed after the XP (extra precision) docking stage. Docking scores were strain corrected with the help of the Strain Rescore script (OPLS3 [47], aqueous solvation model, Energy Offset 0 kcal/mol and default cartesian constraint settings). Subsequently, the dataset was further processed in R [43], where glide docking scores were used to calculate ligand efficiencies as

Ligand efficiency = (Glide docking score − strain penalty) × (N_Heavy atoms_) ^−1^ (1)

and the values obtained normalized. Afterwards, compounds with positive docking scores as well as compounds with strain energy larger than 4 kcal/mol were removed from the dataset. The intersecting set of both obtained lists of docking hits was then considered to contain the putative bimodal compounds. Subsequently, compounds in the lower 0.5 quantile of both normalized ligand efficiencies were excluded. From the resulting list, compounds were selected based on diversity, docking pose, and commercial availability.

### 4.5. AChE Activity Testing

In vitro AChE inhibitory activity was studied using the colorimetric method of Ellman [26] using human recombinant AChE enzyme. To a 96-well microplate, test solutions were applied along with 1 mg/mL bovine serum albumin, 0.5 mM 5,5-dithiobis-(2-nitro-benzoic acid), and 0.5 mM acetylthiocholine iodide. The reaction was initiated by addition of 0.20 units/mL AChE enzyme and followed by colorimetric detection at 405 nm. Experiments were conducted in triplicate. All compounds were dissolved in methanol (maximum 2% methanol at assay conditions that did not affect the enzyme activity) and screened at 200 μM with galantamine as a positive control.

Compounds that fully solubilized in MeOH and exhibited more than 70% inhibition of ACh degradation were further analyzed and their IC_50_ values determined using between five and eight concentrations.

### 4.6. nAChR α7 Activity Testing

#### 4.6.1. Molecular Biology

Human α7 nAChR receptor subunits were cloned and inserted into expression vectors as described previously [53]. Plasmid cDNAs were linearized using a downstream Not I restriction site and purified. cRNA was prepared and capped from the linearized cDNA using the mMessage mMachine T7 transcription kit according to the manufactures protocol. Purified cRNA was aliquoted and stored at a concentration of 0.5 µg·µL^−1^ at −80 °C until further use.

#### 4.6.2. Expression of α7 nAChR in *Xenopus laevis* Oocytes

*Xenopus laevis* oocytes were obtained as described previously [54], briefly, ovary lobes were removed by surgical incision, sliced into small pieces and defolliculated by collagenase treatment. The protocol for this specific study was approved by the Animal Ethics Committee of the University of Sydney (Protocol number: 2013/5915) and carried out according to these guidelines. Stage V and VI oocytes were injected with a total of ~25 ng of cRNA encoding human α7 nAChR with RIC3 (in 5:1 ratio), a protein enhancing the expression of the receptor. Injected oocytes were incubated for 3–5 days at 18 °C in a saline solution (96 mM NaCl, 2 mM KCl, 1 mM MgCl_2_, 1.8 mM CaCl_2_, 5 mM HEPES (hemisodium, pH 7.4)) supplemented with 2.5 mM sodium pyruvate, 0.5 mM theophylline, and 50 µM gentamycin.

#### 4.6.3. Oocyte Electrophysiology

Electrophysiological recordings from *Xenopus laevis* oocytes were performed using the two-electrode voltage-clamp technique as described previously [54,55]. Briefly, oocytes were placed in a custom-built recording chamber and continuously perfused with a saline solution. The saline solution contained 96 mM NaCl, 2 mM KCl, 1 mM MgCl_2_, 1.8 mM CaCl_2_, 5 mM HEPES (hemisodium, pH 7.4). Pipettes were backfilled with 3 M KCl and open-pipette resistances ranged from 0.3 to 1.5 MΩ when submerged in the saline solution. Oocytes were voltage clamped at a holding potential of −60 mV using an Axon Geneclamp 500B amplifier (Molecular Devices, LLC, San Jose, CA, USA). Rapid solution exchange in the oocyte vicinity (order of a few seconds) was ensured by application through a 1.5 mm diameter capillary tube placed approximately 2 mm from the oocyte as described previously [54]. The solution flow rate through the capillary was 2.0 mL/min. Experiments were performed as follows: nAChR currents were initially evoked with three ACh_control_ applications (~EC_20_, 30 µM), a maximum efficacious concentration of ACh_max_ (EC_100_, 3 mM) followed by three additional ACh_control_ applications. Thereafter, test compounds in increasing concentration were applied (25 s), the maximal tested concentration was 200 µM. A wash period of at least 2 min was kept between each application. After the agonist test, new ACh controls were applied (ACh_max_ followed by three additional ACh_control_ applications) and compounds that displayed <1% activation were tested for their ability to modulate the effect of ACh. In these experiments, 10 and 100 µM concentration of a test compound was co-applied with 30 µM ACh. Peak current amplitudes were normalized with respect to the amplitude of current elicited by 3 mM or 30 µM ACh for the agonist and antagonist test, respectively. All experiments were conducted at least in triplicate.

ACh was initially dissolved in milliQ water as 10 mM stock solution. Screened compounds were dissolved as a 50 mM stock solution in DMSO, except for Ýmir-2 and compound **12** which were kept as 10 mM stock solutions. The maximal DMSO concentration in the final dilution did not exceed 2%. This DMSO concentration did not evoke any current from the receptors. Compound dilutions were prepared in a saline solution on the day of the experiment.

### 4.7. Data Analysis

Data analysis was performed as reported previously [24]. Electrophysiological data were analyzed using pClamp 10.2 (Molecular Devices, LLC, San Jose, CA, USA). During analysis, traces were baseline subtracted and responses to individual applications quantified as peak-current amplitudes. Statistical calculations were performed using GraphPad Prism 7 (GraphPad Software, GraphPad, San Diego, CA, USA). Activation of the α7 nAChR was calculated as a percentage of E_max_ response to ACh. For evaluation of the inhibitory activity, the percentage of remaining peak-current amplitudes relative to that of the ACh_control_ (EC_20_) application was calculated. AChE inhibition data were analyzed using GraphPad Prism 7. Absorption readings from the AChE inhibition assay were plotted versus time and linear regression was performed. From the obtained slopes the percentages of inhibition were calculated normalized to the control (Galantamine) and IC_50_ values were determined from non-linear regression analysis. Data were fitted with the slope set to 1 and remaining current amplitude at infinitely high compound concentrations set to 0.

## 5. Conclusions

It was confirmed that HTVS approaches can be applied in the search for novel bimodal drug hits active at AChE and α7 nAChR with good hit rates. Ýmir-2 and Ýmir-10 represent novel compounds with a dual activity profile, i.e., inhibition of AChE and activation of the α7 nAChR, reported for the first time. The successful identification of two bimodal compounds is an encouraging outcome for VS for AD drug hits. Derivatives of Ýmir-2 could be developed into compounds with improved physicochemical properties and activity profiles of clinical interest.

## Figures and Tables

**Table 1:** Docking and in vitro results for tested compounds (**6**–**21**). For AChE, compounds were initially screened at 200 µM followed by IC_50_ measurements for soluble compounds with ≥ 80% inhibition. For α7 nAChR agonism, activities were measured at 200 µM concentrations. For determination of modulation of ACh responses at the α7 nAChR, 100 µM compound was co-applied with 30 µM ACh. Measured data represent the mean ± S.E.M of at least three AChE replicates and independent oocyte experiments.

Compd. ID	Structure	AChE G-Score ^a^ Norm. LE ^b^ %Inhibition ^c,d^ IC_50_ (µM)(pIC_50_ ± SEM)	α7 nAChR G-Score ^a^ Norm. LE ^b^ %Activation ^c^ %Inhibition ^c^	Compd. ID	Structure	AChE G-Score ^a^ Norm. LE ^b^ %Inhibition ^c,d^ IC_50_ (µM) (pIC_50_ ± SEM)	α7 nAChR G-Score ^a^ Norm. LE ^b^ %Activation ^c^ %Inhibition ^c^
**6**	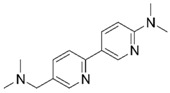	–12.30 –2.11 76.1 ± 0.9% IC_50_ = 58.47 (4.23 ± 0.02)	–13.85 –1.71 0.4 ± 0.4% 47.2 ± 3%	**14**	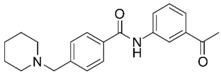	–13.04 –1.06 100% IC_50_ = 1.10 (5.96 ± 0.02)	–15.56 –1.09 0.3 ± 0.3% 60.0 ± 1%
Ýmir-2 (**7**)^f^	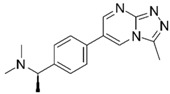	–14.90 –2.63 98.4 ± 0.9% IC_50_ = 2.58 (5.59 ± 0.02)	–11.50 –0.65 7.0 ± 0.9% -	Ýmir-10 (**15**)	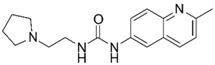	–11.79 –1.18 66.6 ± 1.7% IC_50_ = 114.70 (3.94 ± 0.02)	–13.13 –0.94 2.3 ± 0.4% -
**8**	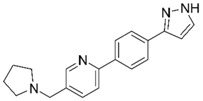	–12.34 –1.19 100%^d^ -	–16.35 –1.60 0.3 ± 0.3% 59.0 ± 5%	**16**	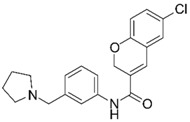	–11.81 –0.50 100%^d^ - -	–16.16 –1.08 1.0 ± 0.0% 90.7 ± 1%
**9**	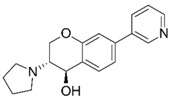	–11.91 –1.23 45.9 ± 3.8%^d^ - -	–15.40 –1.54 1.0 ± 0.6% 66.7 ± 4%	**17**	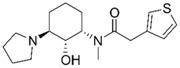	–10.22 –0.59 22.9 ± 1.1% - -	–13.35 –1.00 - -
**10**	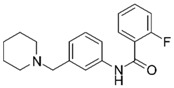	–12.11 –1.10 90.8 ± 0.2% IC_50_ = 11.28 (4.95 ± 0.02)	–15.93 –1.50 0.3 ± 0.3% 85.0 ± 2%	**18**	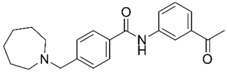	–12.92 –0.86 91.6 ± 2.2% IC_50_ = 17.88 4.75 ± 0.02	–14.49 –0.71 0.7 ± 0.3% 68.7 ± 1%
**11**	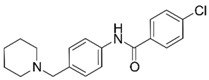	–12.32 –1.18 100%^d^ - -	–15.16 –1.30 0.7 ± 0.3% 73.3 ± 6%	Ýmir-14 (**19**)	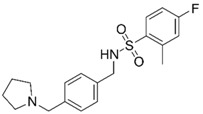	–12.48 –0.88 100% IC_50_ = 1.32 (5.88 ± 0.01)	–13.13 –0.52 0.7 ± 0.3% 96.0 ± 1%
**12**	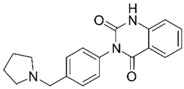	–12.75 –1.14 63.7 ± 4.4%^d^ - -	–15.61 –1.25 0.3 ± 0.3% 81.7 ± 1%	**20** ^f^	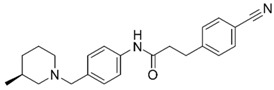	–13.45 –0.87 86.0 ± 0.1% IC_50_ = 33.47 (4.48 ± 0.02)	–13.71 –0.42 0.3 ± 0.3% 94.3 ± 1%
**13**	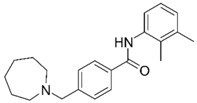	–13.20 –1.12 82.5 ± 1.1% IC_50_ = 30.24 (4.52 ± 0.02)	–15.60 –1.10 0.7 ± 0.7% 68.0 ± 2%	Ýmir-16 (**21**)	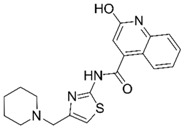	–12.48 –0.71 87.7 ± 0.6% IC_50_ = 7.76 (5.10 ± 0.02)	–13.27 –0.44 0.3 ± 0.3% 97.3 ± 1%

^a^ Glide G-score (kcal/mol); ^b^ Normalized ligand efficiency as defined in Formula 1; ^c^ “-“ indicates “value not determined”; ^d^ % inhibition of supernatant (MeOH); ^e^ galantamine pIC_50_ = 6.64 ± 0.02%, donepezil pIC_50_ = 7.22 ± 0.02; ^f^ tested as racemic mixture.
